# Concomitant heparin use promotes skin graft donor site healing by basic fibroblast growth factor: A pilot prospective randomized controlled study

**DOI:** 10.1016/j.conctc.2024.101375

**Published:** 2024-09-24

**Authors:** Keishi Kohyama, Hisakazu Kato, Hideshi Okada, Takuma Ishihara, Yuji Yasue, Ryo Kamidani, Kodai Suzuki, Takahito Miyake, Hiroshi Okuda, Hirofumi Shibata, Hiroyuki Tomita, Takenori Ogawa

**Affiliations:** a Department of Plastic and Reconstructive Surgery, Gifu University Hospital, Gifu, Japan; b Department of Emergency and Disaster Medicine, Gifu University Graduate School of Medicine, Gifu, Japan; c Center for One Medicine Innovative Translational Research, Gifu University Institute for Advanced Study, Gifu, Japan; d Innovative and Clinical Research Promotion Center, Gifu University Hospital, Gifu, Japan; e Department of Infection Control, Gifu University Graduate School of Medicine, Gifu, Japan; f Department of Otolaryngology-Head and Neck Surgery, Gifu University Graduate School of Medicine, Gifu, Japan; g Department of Tumor Pathology, Gifu University Graduate School of Medicine, Gifu, Japan

**Keywords:** Heparin, Basic fibroblast growth factor, Wound healing, Ointments, Epithelialization

## Abstract

Owing to its mitogenic and angiogenic characteristics, the use of basic fibroblast growth factor (bFGF) to promote wound healing has been investigated. However, its clinical efficacy has fallen short of expectations due to its instability. Heparin has been reported to stabilize bFGF. Therefore, we hypothesized that the combination of these agents would more effectively promote wound healing than bFGF alone; a single-center, two-arm parallel, single-blind, and a prospective randomized controlled pilot study was therefore performed involving 12 patients who underwent split-thickness skin graft harvesting. To ensure a feasible clinical treatment model, commercially available agents were used. The patients were randomly assigned to either the control group treated with bFGF (n = 6) or the intervention group treated with bFGF and heparin (n = 6) in a 1:1 ratio. The wound area and the wound area variation was assessed each week postoperatively, as was the number of days required for epithelialization. As a supplementary analysis, the least-squares means were calculated using a linear mixed-effects model. The results of this study indicate that the combination of bFGF and heparin may more effectively promote wound healing than bFGF alone, consistent with our hypothesis. A multicenter trial based on these data is ongoing.

## Highlights

• Basic fibroblast growth factor exhibits stability in the presence of heparin or heparan sulfate.
• Heparin's effect on improving blood circulation and anti-inflammation promotes wound healing.
• Combination of basic fibroblast growth factor and heparin effectively promotes wound healing.
• Drugs in an ointment form demonstrate high versatility and are practical in clinical settings.

## 1 Introduction

With an aging population, the incidence of chronic skin ulcers, such as leg ulcers and pressure injuries, is increasing. This trend poses a significant challenge as these conditions can profoundly impact a patient's long-term quality of life, necessitating substantial resources in terms of human capital, goods, and time. Consequently, it imposes a financial strain on the healthcare system [1]. The primary strategy for addressing this problem is early wound healing. Furthermore, although local wound treatment represents the basis of all approaches, advancements in this area have been limited. Conversely, research on the wound healing process has progressed, and the complex interplay among various factors has become more apparent. One such notable factor is basic fibroblast growth factor (bFGF). Owing to its mitogenic and angiogenic characteristics, bFGF induces neovascularization, tissue remodeling, and wound healing, as shown by several studies [2]. Therefore, local bFGF administration at the wound site was expected to promote wound healing in clinical settings. However, a clinical pilot study in the United States showed no effect of bFGF on wound healing [3]. Currently, bFGF is used for the treatment of wounds in only a few countries, including Japan; reports from these countries on the clinical usefulness of bFGF [1,4] have shown some benefits but no remarkable effects and, therefore, have not led to renewed interest in bFGF. The instability of bFGF has been suggested as a rationale for its inefficacy in clinical practice [[5], [6], [7]].

bFGF is stabilized by heparin [[5], [6], [7], [8], [9], [10]], and clinical reports suggest that such a combination improves its efficacy [9]. Hence, it is assumed that the combined use of bFGF and heparin will enhance the therapeutic impact of bFGF in wound healing. However, there is a lack of clinical reports regarding the promotion of wound healing through combined treatment with bFGF and heparin. Presently, topical bFGF formulations are approved and marketed in Japan for the promotion of wound healing. Conversely, topical heparin ointments are approved and marketed for their anti-inflammatory properties in the treatment of blood circulation disorders. The combination of bFGF and heparin is clinically feasible when both topical agents are used together, and we hypothesized that this combination would promote wound healing to a greater extent than bFGF treatment alone. The present study is a pilot study conducted as the first step to investigate the wound-healing effects of the combination of bFGF and heparin. Therefore, we used a combination of both topical agents as a model for the combined use of bFGF and heparin, which is currently feasible in clinical practice. The chosen wounds for treatment were split-thickness skin graft (STSG) donor sites, which are presently managed with bFGF formulation at our institute.

## 2 Materials and Methods

### 2.1 Study design

This study is a two-arm parallel, assessor-blinded, prospective randomized controlled clinical pilot study designed to investigate the effectiveness of a mixed ointment containing bFGF and heparin on STSG donor sites. The pilot study aimed to assess the potential of this mixed ointment as a novel therapy to expedite wound healing. Additionally, this investigation served as a data-collection phase in preparation for the upcoming prospective randomized controlled trial. This study was conducted between November 2020 and November 2021 at the Department of Plastic and Reconstructive Surgery at Gifu University Hospital (Gifu, Japan). The study is reported according to the CONSORT reporting guidelines [11].

### 2.2 Ethics approval

This study was conducted in accordance with the ethical principles embodied in the 1975 Declaration of Helsinki. The study protocol was reviewed and approved by the Research Ethics Committee of Gifu University Hospital (approval no.: 2020–120) and was registered with the Japan Registry of Clinical Trials (jRCT1041230082; Title: Effect of topical treatment with a combination of basic fibroblast growth factor and heparin on skin graft donor site healing: a randomized controlled pilot study; URL: https://jrct.niph.go.jp/en-latest-detail/jRCT1041230082). All patients enrolled in this study provided written informed consent and were aware that they could withdraw from the study at any time without needing an explanation. Participation in or withdrawal from the study did not affect the treatment process in patients, and anonymity was guaranteed.

### 2.3 Participants and sample size

Patients aged ≥20 years who signed an informed consent form, underwent STSG harvesting from the thigh to prepare for other plastic surgeries for which the skin was needed and had complete data were included in the study. Patients who had been previously enrolled in the study or showed bleeding tendencies were excluded. A planned sample size of 12 patients was determined based on the number of participants required to assess the feasibility of the study method.

### 2.4 Materials

**bFGF formulation.** Genetically engineered recombinant human bFGF kit (Fibrast Spray®, Kaken Pharmaceutical Co., Ltd., Tokyo, Japan [CAS number 131094-16-1]), which comprised freeze-dried bFGF and benzalkonium chloride solution, was used. This clinical-grade formulation is used for wound care in humans in Japan. According to the manufacturer's instructions, 100 μg freeze-dried bFGF was dissolved in 1 mL benzalkonium chloride solution. As the recommended concentration was 30 μg bFGF per 30 cm^2^ area (1 μg/cm^2^), 300 μL bFGF solution was sprayed over a 30 cm^2^ wound from a 5 cm distance.

**Heparin.** An ointment containing 500 units/g heparin with purified lanolin as the base (Heparin Z® ointment, Zeria Pharmaceutical Co., Ltd., Tokyo, Japan [CAS number 9041-08-1]) was used. The specific gravity of purified lanolin ranged from 0.932 to 0.945 g/cm^3^; thus, the specific gravity of this ointment was similar. This clinical-grade ointment is used in Japan to treat pain and inflammatory conditions caused by blood circulation disorders in humans.

**Preparation of topical formulation mixed with bFGF and heparin.** Assuming that the thickness of the ointment applied was 3 mm, the bFGF concentration was set at 1 μg/0.3 cm^3^ to spread at a concentration of 1 μg/cm^2^ on the wound surface. The topical formulation (ointment G) was prepared by mixing freeze-dried bFGF dissolved in benzalkonium chloride solution with Heparin Z® ointment to achieve a bFGF concentration of 1 μg/0.3 cm^3^. The heparin concentration at the time of application was 137.5–139.4 units/cm^2^.

### 2.5 Interventions

Eligible patients were recruited before surgery, and informed consent was obtained from all patients. Patients were randomly allocated to either the control or intervention group at a 1:1 ratio before surgery. The control group, consisting of six patients, underwent standard treatment with bFGF and white petrolatum (Propeto®, Maruishi Pharmaceutical Co., Ltd., Osaka, Japan), while the intervention group, also comprising six patients, received treatment with ointment G. Although not adopted worldwide, bFGF has been reported to not only heal wounds faster but also alleviate scars [12]. Therefore, we adopted bFGF formulation as a standard treatment of many wounds, including STSG donor sites.

After general anesthesia was administered, donor sites were prepared with a 10 % povidone-iodine solution (Povidone-Iodine Solution [Meiji], Meiji Seika Pharma Co., Ltd., Tokyo, Japan). Experienced plastic surgeons obtained all STSGs from the lateral thigh at a depth of 0.25 mm using an air-powered dermatome (Air Dermatome®, Zimmer Biomet, Warsaw, USA). Immediately after skin harvesting, donor sites were covered with gauze soaked in an epinephrine-saline solution (100 mL 0.9 % saline [Isotonic Sodium Chloride Solution, Hikari Pharmaceutical Co., Ltd., Tokyo, Japan], 1 mg epinephrine [Adrenaline injection 0.1 %, Terumo Corp., Tokyo, Japan]). After achieving complete hemostasis, donor sites were covered with a chitin film (Beschitin W®, Nipro Corporation, Osaka, Japan). The covering film was thoroughly soaked in saline on the first postoperative day and was carefully removed. Patients with minimal bleeding were closely monitored until complete hemostasis was achieved. Subsequently, the allocated treatment was administered. Wounds were observed and treated daily. Daily treatment of STSG donor sites could increase pain, induce bleeding, and impede healing. Therefore, treatment was performed as slowly as possible. To prevent adhesions between wound and dressing, the dressing was applied as follows. In the control group, the wound was sprayed with the bFGF formulation solubilized in benzalkonium chloride, as described above, and was left for 30 s. White petrolatum was applied to a silicone-faced wound dressing (SI-Aid®, ALCARE Co., Ltd., Tokyo, Japan). In the intervention group, ointment G was applied to the wound, followed by the application of the silicone-faced wound dressing. The dressing in each group was secured using elastic adhesive gauze, and an absorbent dressing pad was applied, depending on the amount of exudate. Following the initial dressing change, the plastic surgeons provided daily treatment at the donor sites in the same manner. The study was terminated if complications (e.g., excessive bleeding from the wound) occurred during the study or if patients withdrew their consent. All patients received routine medications and standard care, independent of the type of donor site treatment. The follow-up period was up to 8 weeks post-operation.

### 2.6 Outcome measurements

In this study, the outcomes measured included the proportion of the wound area at 1, 2, 3, and 4 weeks postoperatively, the wound area variation at each week, and the number of days required for complete epithelialization.

### 2.7 Data collection procedures

The following data were collected from medical records at the time of patient enrolment in the study: age, sex, body mass index (BMI), diagnosis, comorbidities, and hematological data. The wound area at the donor sites was measured intraoperatively and at 1, 2, 3, and 4 weeks postoperatively; two experienced plastic surgeons thoroughly inspected and measured the wound area at each timepoint. Additionally, measurements were taken as needed; if a measurement could not be taken on the scheduled date, it was taken on the nearest available date.

Prior to measurement, wounds were washed with warm saline; the ointment was removed. The wound area was copied by placing a transparent plastic film sheet over the wound surface and tracing the boundary of the non-epithelialized area. The non-epithelialized areas tended to be wet and thereby easily adhered to the plastic film sheet. In addition, two plastic surgeons, different from the surgeon, also evaluated the wound grossly. Based on these results, the boundary of the non-epithelialized area was comprehensively determined. The area was measured using a planimeter (X-Plan 360 dII®, Kantum Ushikata Co., Ltd., Yokohama, Japan) from a plastic film sheet with the copied wound area. Minor wounds and epithelialized areas were measured whenever possible. Complete epithelialization was defined as continuous coverage of the epidermis with no remaining scabs.

### 2.8 Allocation sequence generation and concealment

An independent researcher from a different institution used block randomization to randomly allocate the participants to either the control or intervention group in a 1:1 ratio, using a random sequence generated in Microsoft Excel (Microsoft Excel 2019, Microsoft Corp., Redmond, USA) with a block size of 4. The resulting list was enclosed in sealed, opaque envelopes. Participants were recruited separately from the randomization process, ensuring adequate allocation concealment. As both treatments had distinct features and could be recognized by the study team, this study was single-blinded. However, the data analysis was blinded.

### 2.9 Statistical analysis

The sample size was set to 12 patients to ensure the feasibility and accuracy of mean and variance estimation for the outcomes [13]. Patient characteristics are expressed as mean ± standard deviation (SD) for continuous variables and as counts and percentages for categorical variables. Study outcomes are presented as mean ± SD. The least-squares means of the wound area and wound area variation were calculated using a linear mixed-effects model, assuming compound symmetry in the correlation structure. The analysis was initially designed with the assumption of an autoregressive (AR1) correlation structure; however, this assumption was modified to the above because it was impossible to estimate. All analyses were performed using R software version 4.2.2 (www.r-project.org).

## 3 Results

### 3.1 Patient characteristics

**Fig. 1:**
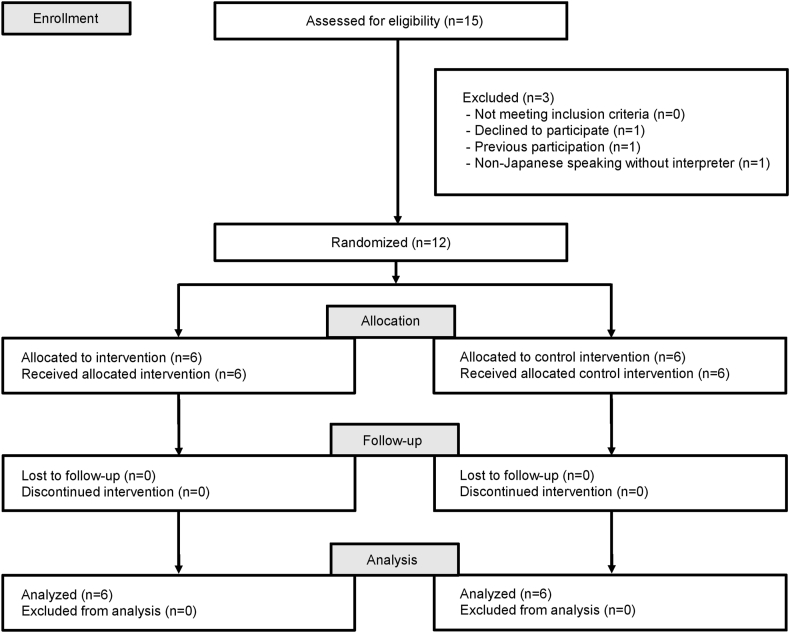
CONSORT flow diagram of study participants.

Three participants were excluded from the initial pool of 15 eligible patients because of declining to participate, previous participation, or non-Japanese speaking without an interpreter. The remaining 12 patients were randomly assigned to either the control group (*n* = 6) or the intervention group (*n* = 6). During the study period, none of the study participants used steroids or antitumor drugs or dropped out of the study. Finally, data analysis was conducted with all 12 participants (Fig. 1).

There were eight females and four males, with a mean age of 63.4 ± 12.8 years (range: 33–77 years) and mean BMI of 23.7 ± 5.1 kg/m^2^ (range: 13.3–31.3 kg/m^2^). The participant characteristics stratified by treatment are summarized in Table 1. Five patients (41.7 %) had cardiovascular disease as a comorbidity, whereas one patient (8.3 %) presented with renal failure and another patient (8.3 %) had diabetes. Among the diagnoses requiring reconstructive surgery with skin grafts, malignancy was observed in 11 patients (91.7 %) and chronic skin ulcers in one patient (8.3 %). The wound area at donor sites measured 31.14–119.34 cm^2^ (mean: 65.76 ± 24.50 cm^2^). Statistical significance testing for differences was not conducted since patients were randomly assigned to groups, and some imbalance could be expected in a pilot study.

**Table 1:** Participants’ characteristics stratified by treatment.

Variable		Overall	Control	Intervention
		*N* = 12	*N* = 6	*N* = 6
Sex:	Female	8 (66.7 %)	4 (66.7 %)	4 (66.7 %)
	Male	4 (33.3 %)	2 (33.3 %)	2 (33.3 %)
Mean age at surgery, years (range)		63.4 (33–77)	58.0 (33–74)	68.8 (55–77)
Mean body mass index at surgery, kg/m^2^ (range)		23.7 (13.3–31.3)	25.8 (20.5–31.0)	21.6 (13.3–31.3)
Patient history:	Cardiovascular disease	5 (41.7 %)	1 (16.7 %)	4 (66.7 %)
	Renal failure	1 (8.3 %)	0 (0.0 %)	1 (16.7 %)
	Diabetes mellitus	1 (8.3 %)	0 (0.0 %)	1 (16.7 %)
Cause of surgery:	Malignancy	11 (91.7 %)	6 (100.0 %)	5 (83.3 %)
	Chronic skin ulcer	1 (8.3 %)	0 (0.0 %)	1 (16.7 %)
Mean wound area at donor sites, cm^2^ (range)		65.8 (31.1–119.3)	63.9 (44.1–91.4)	67.7 (31.1–119.3)

### 3.2 Outcomes

**Fig. 2:**
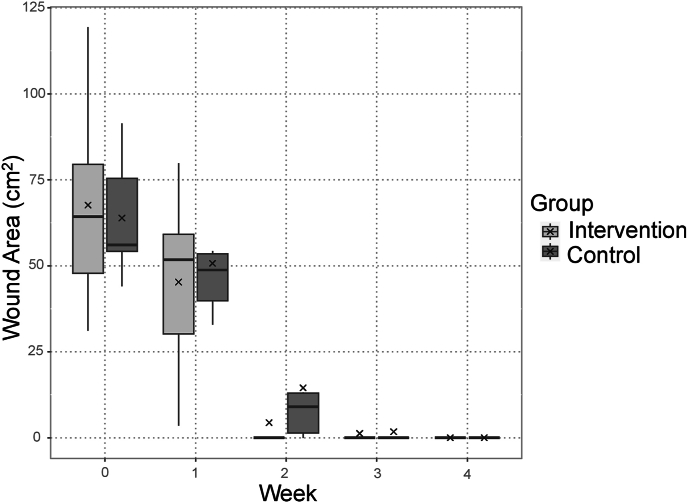
Changes in the wound area over time based on the aggregate results for the wound area. × : mean.

**Fig. 3:**
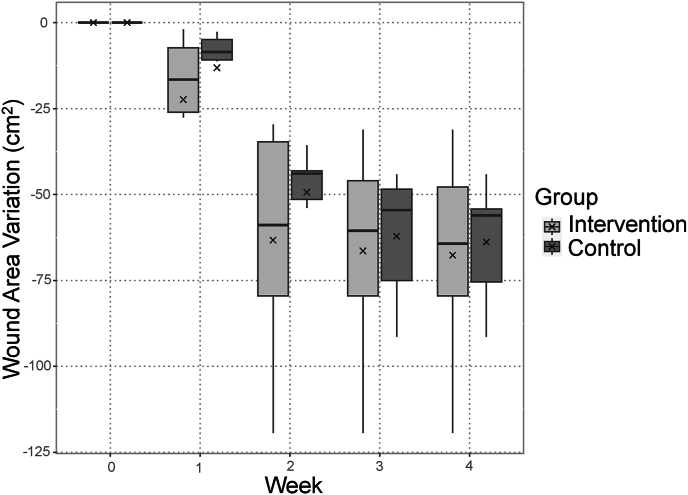
Changes in the wound area variation over time based on the aggregate results for the wound area. × : mean.

Removing the chitin film, several patients experienced slight petechial bleeding and mild pain. However, no further abnormal bleeding, infection, or other adverse events were observed in the study cohort. The mean postoperative wound area measured 63.9 (100 %) ± 18.4 cm^2^ in the control group and 67.7 (100 %) ± 31.2 cm^2^ in the intervention group. The mean postoperative wound area improved to 50.7 (79.4 %) ± 17.2 cm^2^ and 45.3 (66.9 %) ± 27.3 cm^2^ at 1 week postoperatively, 14.5 (22.7 %) ± 21.0 cm^2^ and 4.4 (6.5 %) ± 10.7 cm^2^ at 2 weeks postoperatively, 1.7 (2.7 %) ± 4.2 cm^2^ and 1.2 (1.8 %) ± 3.0 cm^2^ at 3 weeks postoperatively, and 0 cm^2^ (0 %) and 0 cm^2^ (0 %) at 4 weeks postoperatively in the control and intervention groups, respectively. The mean wound area variation was −13.2 ± 15.4 cm^2^ and -22.4 ± 23.4 cm^2^ by 1 week postoperatively, −49.4 ± 14.2 cm^2^ and -63.3 ± 34.8 cm^2^ by 2 weeks postoperatively, −62.1 ± 19.6 cm^2^ and -66.4 ± 31.9 cm^2^ by 3 weeks postoperatively, and −63.9 ± 18.4 cm^2^ and -67.7 ± 31.2 cm^2^ by 4 weeks postoperatively in the control and intervention groups, respectively (Table 2). As a supplementary analysis, the least-squares means of the wound area and wound area variation were calculated using a linear mixed-effects model, and the results are presented in Table 3. A graph depicting the change in wound area over time is presented in Fig. 2, whereas a graph depicting the change in wound area variation over time is presented in Fig. 3. Both were based on the primary analysis. The mean number of days required for complete epithelialization was 16.5 ± 4.4 days in the control group and 12.8 ± 5.6 days in the intervention group. Given the nature of the pilot study, no analysis was conducted to assess differences.

**Table 2:** Primary analysis: aggregate results for wound area and wound area variation.

Characteristic		Overall	Control	95 % CI	Intervention N = 6 Mean (SD)	95 % CI	*p*-value (Welch's two-sample *t*-test)
		*N* = 12	*N* = 6				
		Mean (SD)	Mean (SD)	Lower, Upper		Lower, Upper	
Wound area:	Baseline	65.8 (24.5)	63.9 (18.4)	45, 83	67.7 (31.2)	35, 100	0.8
	At 1 week postoperatively	48.0 (22.0)	50.7 (17.2)	33, 69	45.3 (27.3)	17, 74	0.7
	At 2 weeks postoperatively	9.4 (16.8)	14.5 (21.0)	−7.5, 37	4.4 (10.7)	−6.9, 16	0.3
	At 3 weeks postoperatively	1.5 (3.5)	1.7 (4.2)	−2.7, 6.2	1.2 (3.0)	−2.0, 4.4	0.8
	At 4 weeks postoperatively	0.0 (0.0)	0.0 (0.0)	N.C.	0.0 (0.0)	N.C.	N.C.
Wound area variation:							
	By 1 week postoperatively	−17.8 (19.5)	−13.2 (15.4)	−29, 3.0	−22.4 (23.4)	−47, 2.1	0.4
	By 2 weeks postoperatively	−56.3 (26.4)	−49.4 (14.2)	−64, −34	−63.3 (34.8)	−100, −27	0.4
	By 3 weeks postoperatively	−64.3 (25.3)	−62.1 (19.6)	−83, −42	−66.4 (31.9)	−100, −33	0.8
	By 4 weeks postoperatively	−65.8 (24.5)	−63.9 (18.4)	−83, −45	−67.7 (31.2)	−100, −35	0.8

SD, standard deviation; CI, confidence interval; N.C., not calculated.

**Table 3:** Supplementary analysis: least-squares means of the wound area and wound area variation using a linear mixed-effects model.

Characteristic		Control	95 % CI	Intervention	95 % CI
		*N* = 6 LSM	Lower, Upper	*N* = 6 LSM	Lower, Upper
Wound area:	At 1 week postoperatively	49.32	38.31, 60.33	43.16	32.04, 54.28
	At 2 weeks postoperatively	13.11	2.10, 24.12	2.29	−8.84, 13.41
	At 3 weeks postoperatively	0.34	−10.67, 11.35	−0.85	−11.98, 10.27
	At 4 weeks postoperatively	−1.39	−12.40, 9.63	−2.10	−13.22, 9.03
Wound area variation:	By 1 week postoperatively	−7.13	−18.14, 3.88	−13.29	−24.41, −2.17
	By 2 weeks postoperatively	−43.34	−54.35, −32.33	−54.16	−65.29, −43.04
	By 3 weeks postoperatively	−56.11	−67.12, −45.10	−57.30	−68.43, −46.18
	By 4 weeks postoperatively	−57.84	−68.85, −46.82	−58.55	−69.67, −47.42

LSM, least-squares mean; CI, confidence interval.

## 4 Discussion

In this pilot study, topical treatment of clinical wounds with a combination of bFGF and heparin was performed. Treatment of STSG donor sites with a topical formulation of bFGF and heparin was compared to treatment with the conventional bFGF formulation alone. The study results indicated that the combination of bFGF and heparin may promote wound healing more effectively than bFGF alone, which is consistent with our hypothesis.

### 4.1 bFGF

In this study, the key process in wound healing was epithelialization, which depends on the ability of keratinocytes (the major epithelial cells in the epidermis [14]) to migrate, proliferate, and differentiate [15]. bFGF, discovered in 1974, is a well-characterized member of the FGF family of molecules with established angiogenetic effects [16]. It plays a crucial role in the activity and migration of keratinocytes [10]. A single application of recombinant human bFGF at the time of injury accelerates the rate of epithelization by 20 % [17].

Furthermore, bFGF potently modulates cell proliferation, motility, differentiation, and survival [9] and also plays a crucial role in diverse cellular functions, including embryonic development, angiogenesis, tissue regeneration, bone regeneration, nervous system development, and maintenance, stem cell self-renewal, and wound healing [5,[15], [16], [17], [18], [19], [20]]. A decrease in the concentrations of growth factors, including bFGF, in chronic wounds and ischemic conditions inhibits these cellular processes, thereby preventing healing and angiogenesis [21]. While inflammatory processes mobilize macrophages and mast cells that secrete endothelial growth factors (such as bFGF) [22] or chemotactic factors [14], exogenous growth factors can accelerate wound healing [23]. Reports indicate improved fibroblast proliferation and DNA synthesis via ERK/Akt phosphorylation [2], activation of local macrophages up to the remodeling phase [22], and enhanced expression of cytokines such as vascular endothelial growth factor (VEGF) and transforming growth factor-β (TGF-β) [10]. VEGF can enhance endothelial permeability, capillary fusion, and macro-angiogenesis, whereas TGF-β can facilitate on-site cell immigration and proliferation [10].

### 4.2 Challenges of using bFGF as a therapeutic agent

The instability of bFGF has been identified as one of the reasons why its expected effects have yet to be achieved in clinical practice [[5], [6], [7]]. When exogenous bFGF is directly administered to the wound site, it undergoes rapid degradation and inactivation by numerous proteolytic enzymes after diffusing into the wound, resulting in a very short half-life [10,24,25]. Thus, addressing its instability could be crucial for its clinical utilization.

### 4.3 Heparin as a countermeasure

A key candidate for addressing this issue is the combined use of bFGF and heparin, which is a sulfated mucopolysaccharide recognized for its natural anticoagulant in animals [26]. While heparin is renowned for its anticoagulant effect, the focus has broadened to include a wide range of biological activities over recent years, including anti-inflammatory effects, activation of lipoprotein lipase, growth factor activation, and immune modulation [[27], [28], [29]]. bFGF, a heparin-binding protein, has been reported to exhibit stability in the presence of heparin or heparan sulfate (HS) [[5], [6], [7],^9^,^10^], with multiple possible mechanisms underlying this phenomenon. In particular, the binding mainly occurs via spatially matching electrostatic interactions between the negatively charged N- and O-sulfated groups of heparin and the basic lysine and arginine residues of bFGF [30,31]. This binding has been demonstrated to slow down the diffusion of bFGF [20,32] and protect it from inactivation caused by factors like heat, acid, non-enzymatic glucosylation [33,34], and proteases such as trypsin and plasmin [33].

Heparin is often considered an analog of HS because both glycosaminoglycans are composed of the same monomeric subunits, and heparin is regarded as a more sulfated, tissue-specific HS variant [35]. Both heparin and HS can interact with several proteins [[36], [37], [38]], and heparin is often employed experimentally as a proxy for HS due to their structural similarity [35]. Several studies on various cell types suggest that HS glycosaminoglycans are necessary for the stable binding of the FGF ligand to the receptor tyrosine kinase and for sustained signaling [5,39]. Thus, to a varying degree, heparin is expected to have a similar direct effect on bFGF. While the direct effect on bFGF is not immediately apparent, the removal of putative heparin- or HS-binding regions from the growth factor dramatically diminishes its biological activity [40]. Furthermore, heparin acts as a cofactor that promotes the binding of bFGF to high-affinity receptors, thereby enhancing its bioactivity [9,23,41].

Overall, the combination of bFGF and heparin is expected to enhance the wound-healing effect. Animal studies have previously reported good results [6]; however, these results have not yet been successfully translated to the clinical setting.

### 4.4 Potential additional effects of heparin

Heparin is thought to play a significant role as a cofactor and modulator of bFGF expression. Nonetheless, the biological activity of heparin may also affect wound healing. Its strong anticoagulant effect prevents microcirculation thrombosis [10], whereas its chemotactic effect on endothelial cells stimulates neovascularization [29], thus improving local blood circulation. Additionally, heparin reduces inflammation by binding to and sequestering chemokines at the wound site [10]. Collectively, these effects may contribute to promoting wound healing. Heparin can bind to cytokines and other growth factors, such as amphiregulin and keratinocyte growth factor, especially in keratinocytes, thereby modifying their growth-inducing effects [[42], [43], [44]].

However, it has also been identified as a potent inhibitor of cell proliferation in various cell types [45]. In keratinocytes, it not only hinders growth and adherence but also binds and enhances the growth-inhibitory function of tumor necrosis factor-α [42]. Although the predominant system in wound healing is complex, there have been reports of the reversal of growth inhibition to growth stimulation by its concentration in skin organ cultures [46]. Therefore, it is unlikely and has not been reported, that heparin alone significantly improves wound healing.

### 4.5 Risk of bleeding

Heparin poses a significant potential risk of severe bleeding complications due to its strong anticoagulant activity [9,47,48]. Direct administration to the wound could potentially induce bleeding. Therefore, in this study, vigilant and proactive measures were taken to prevent bleeding; however, bleeding did not occur as anticipated. While the clinical risk appears to be low for the methods and target wounds used in this study, verification is nonetheless necessary.

### 4.6 Alternatives to heparin usage

Alternative approaches to address the instability of bFGF involve using gel or some scaffolds as vehicles for a slow-release mechanism [8,[49], [50], [51], [52], [53]]. From this perspective, the ointment form of the mixed formulation used in this study has several advantages. The ointment, in this study, exhibited thixotropic properties (close to sol) and could transition into either a gel or sol state. The gel, being less fluid than the sol, could adhere to the wound for a specific duration. Gels, when combined with slow-release agents, have the potential to not only improve wound healing efficacy but also simplify procedures and reduce healthcare costs. However, in clinical practice, prolonged application of the gel poses the risk of infection and may lead to a misjudgment of changes in the treatment strategy. This is particularly relevant for chronic wounds, a prevalent social problem that typically originates from tissue necrosis based on underlying conditions. These wounds often occur in areas prone to contamination, such as the lower extremities and buttocks. Furthermore, once a wound develops, it does not necessarily follow a linear healing trajectory; there is often a combination of ongoing healing and new developments. The STSG donor sites in this study and artificially created wounds in animal experiments differ significantly from clinical situations. These wounds are relatively clean in terms of their development and course. Therefore, in the overall treatment process, the effectiveness of using a gel to enhance wound healing or reduce healthcare costs remains uncertain. This uncertainty may contribute to the limited advancement in clinical applications. In contrast, the approach of wound cleaning, observation, and daily treatment proves to be a safe and reliable practice. Drugs in an ointment form suitable for this purpose demonstrate high versatility and are practical in clinical settings. Additionally, since keratinocyte migration thrives in a fluid environment [14], the thixotropic property, which maintains a degree of fluidity, may offer advantages.

### 4.7 Limitations

This study has some limitations. First, the study was non-matched and included variations in patient backgrounds and wound sizes. Factors such as diabetes mellitus, age, excessively abnormal BMI, poor nutritional status, and limb ischemia due to bloodstream disorders, which may impact wound healing, should be considered. In this study, of the 12 patients, 11 (91.7 %) had head and neck cancer; most of them had some type of eating disorder. Therefore, poor nutritional status could not be ruled out. In addition, eight (66.7 %) patients had some comorbidity. These may have adversely affected the healing time in this study. Second, the wound area measurement was based on gross findings, which may not always be consistent with pathologic epithelialization. Third, the concentrations of bFGF and heparin in this study and the ointment base used strongly depended on the status of commercially available products. Neither of the topical agents is available worldwide as a standard drug. Administering the liquid heparin or using the bFGF formulation for the wounds other than by the prescribed method of spraying was also impossible. Therefore, white petrolatum was used in the control group to create a wound environment similar to that of lanolin, the main ointment base in the experimental group. However, the wound environment was not exactly the same. Previous studies have reported a bell-shaped dose-effect relationship for bFGF or heparin [54,55], and the parameters of the drugs and ointment base can potentially be further optimized for enhanced wound healing. The extent to which the combination of heparin improved bFGF instability needs clarification as it is directly related to the method of use and frequency of treatment. Although treatment was administered once a day in accordance with the accompanying documentation for bFGF, the frequency of treatment may be reduced with improved stability. However, given that heparin is susceptible to *in vivo* degradation and desulfation by heparinases and has a short half-life [56], achieving this may be challenging. Finally, there are limitations to this wound model. Since this study included an acute model of partial thickness wounds for pilot study, the results cannot not be extrapolated to target chronic wounds. However, chronic wounds are featured by elevated levels of proteases and heightened proteolytic activity [57]. In such an environment, heparin may be more effective in protecting bFGF from proteases. In this model, bFGF might likely not be very effective except for its epithelial migration effect. In deeper wounds, the angiogenic and other properties of bFGF may be more effective.

## 5 Conclusion

The findings of this study suggest that the combination of bFGF and heparin may promote wound healing at STSG donor sites more effectively than bFGF alone. Since this is a pilot study, further investigations are needed to confirm these results. A multicenter prospective randomized controlled clinical trial based on these data is currently ongoing. Considering the limitations of research, animal studies are being contemplated, and adjustments to the utilized drugs and ointment base components are anticipated to further optimize wound healing outcomes. The results of this study suggest that the use of bFGF in wound healing, which was almost abandoned, should be revisited, providing a new strategy for the local treatment of wounds addressing an area where progress has encountered obstacles. Furthermore, as the products used here are commercially available and already in clinical use, the challenges associated with implementing this treatment in clinical settings should be minimized, potentially allowing for its swift adoption.

## Funding

This research did not receive any specific grant from funding agencies in the public, commercial, or not-for-profit sectors.

## Data availability

The data that support the findings of this study are available from the corresponding author upon reasonable request.

## CRediT authorship contribution statement

**Keishi Kohyama:** Writing – original draft, Visualization, Methodology, Investigation, Conceptualization. **Hisakazu Kato:** Writing – review & editing, Data curation. **Hideshi Okada:** Writing – review & editing, Project administration, Methodology, Conceptualization. **Takuma Ishihara:** Writing – review & editing, Visualization, Formal analysis. **Yuji Yasue:** Writing – review & editing, Investigation. **Ryo Kamidani:** Writing – review & editing, Data curation. **Kodai Suzuki:** Writing – review & editing, Data curation. **Takahito Miyake:** Writing – review & editing, Investigation. **Hiroshi Okuda:** Writing – review & editing, Investigation. **Hirofumi Shibata:** Writing – review & editing, Investigation. **Hiroyuki Tomita:** Writing – review & editing, Supervision. **Takenori Ogawa:** Writing – review & editing, Supervision.

## Declaration of competing interest

The authors declare that they have no known competing financial interests or personal relationships that could have appeared to influence the work reported in this paper.

## Data Availability

Data will be made available on request.
